# Effect of pediatric mouthwashes on the color stability of dental restorations with composite resins. *In vitro* comparative study

**DOI:** 10.4317/jced.532747

**Published:** 2024-11-01

**Authors:** Lizbeth Martinez-Ccahuana, Evelyn Álvarez-Vidigal, Luis-Ernesto Arriola-Guillén, Denisse Aguilar-Gálvez

**Affiliations:** 1 Division of Pediatric Dentistry, School of Dentistry, Universidad Científica del Sur, Lima, Perú; 2 Division of Orthodontics, School of Dentistry, Universidad Científica del Sur, Lima, Perú

## Abstract

In this article by Martinez-Ccahuana and colleagues (J Clin Exp Dent. 2022;14(11):e897-902), there is an error in the authors of the manuscript. The correct author list is: Lizbeth Martinez-Ccahuana, Evelyn Álvarez-Vidigal, Luis-Ernesto Arriola-Guillén, Denisse Aguilar-Gálvez. References 1. Lizbeth Martinez-Ccahuana, Evelyn Álvarez-Vidigal, Luis-Ernesto Arriola-Guillén, Denisse Aguilar-Gálvez. Effect of pediatric mouthwashes on the color stability of dental restorations with composite resins. In vitro comparative study. J Clin Exp Dent. 2022;14(11):e897-902. PMid: 36458028 PMCid: PMC9701350.

